# The London Lancet Describes the Homœopathic Physician (Extract)

**Published:** 1882-12

**Authors:** 


### THE LONDON LANCET DESCRIBES THE HOMŒOPATHIC PHYSICIAN.

The *London Lancet*, in a recent issue, in an article headed "Quackery within the Profession," says, "Nothing is so much needed just now as the rise in our midst of a strong and uncompromising apostle of sincerity in science—a man of unpitying animosity to humbug in all its forms, who will not hesitate, at any bidding, to denounce wrong-doing and untruthfulness, let who may be the offender. It is time that a spirit of manliness went out in our ranks to chase away the lying spirit of mock courtesy—the faint-hearted and time-serving sentimentality which makes us so ready to look kindly on any pretender and so reluctant to expose any pretense."—*Breyfogle's Address.*

